# Knowledge, Awareness, and Practice of Pediatricians Regarding Infant Oral Health Care and Early Childhood Caries in the State of Assam, India

**DOI:** 10.7759/cureus.52060

**Published:** 2024-01-10

**Authors:** Bhaskar Das, Sandeep Barman, Amal Baishya, Ramen Haloi, Dipshikha Das

**Affiliations:** 1 Department of Dentistry, Diphu Medical College and Hospital, Diphu, IND; 2 Department of Pediatrics, Nagaon Medical College and Hospital, Nagaon, IND; 3 Department of Psychiatry, Diphu Medical College and Hospital, Diphu, IND; 4 Department of Public Health Dentistry, Government Dental College, Dibrugarh, IND

**Keywords:** nursing bottle caries, streptococcus mutans, pediatrician, infant oral health care, early childhood caries

## Abstract

Background and aim: Pediatricians are the first point of contact for evaluating a child’s health. Hence, our study was done to evaluate the knowledge, awareness, and practice of pediatricians regarding infant oral health care and early childhood caries (ECC) in the state of Assam.

Methods: A close-ended questionnaire was circulated among the pediatricians of Assam. Data regarding knowledge, awareness, and practice involving the oral health of children and ECC was collected.

Results: A total of 110 pediatricians participated in the study and the data obtained was subjected to chi-square analysis. Most of the participants (n=64, 67%) displayed adequate knowledge regarding dental caries and the way to identify them. Although most of the participants knew about practices leading to dental caries, they were lacking in knowledge regarding the deleterious effect of bottle feeding and related habits 65 (50-60%). Also, most of the participants (n=69, 62%) failed to educate the caregivers of the child regarding early dental visits.

Conclusion: The majority of pediatricians displayed adequate knowledge regarding dental caries and their prevention but failed to educate the parents and caregivers of children regarding the importance of first dental visits and the maintenance of oral health.

## Introduction

The American Academy of Pediatric Dentistry (AAPD) defined early childhood caries (ECC) as the presence of one or more decayed (non-cavitated or cavitated lesions), missing (due to caries), or filled tooth surfaces in any primary tooth in a child at ≤71 months of age (2019) [1].

Dental caries is one of the most common diseases affecting children, i.e., five times more common than asthma and seven times more common than hay fever in children [2,3]. Statistically, 67% of the children would have experienced dental decay by the age of 53. The 2017 Global Burden of Disease study reported approximately 532 million untreated dental caries cases in deciduous teeth. A recent systematic review in India revealed a pooled prevalence of ECC of 46.9% and that one in every two children in India is suffering from ECC, reflecting the grievous state of the disease in India [4].

Oral health is one of the most neglected aspects of general health [4]. ECC, when left untreated, can severely undermine the quality of life of the child as they can present with difficulty in chewing, eating, speaking, and attending school [5,6].

Carious lesions in early childhood are characterized by rapidly progressing smooth surface lesions, primarily initiating from the upper incisors and progressively involving the first primary molars in both jaws due to the sequence of eruption. The canines and second molars have less opportunity to be affected due to their eruption at a later stage, but they do eventually get involved in cases of neglected oral health [7]. Caries in infants and children can lead to various sequelae if left untreated, such as severe tooth pain, abscess, destruction of bones, the spread of infection via the bloodstream, etc. [8].

Children less than three years old are seldom seen routinely by the dentist, but they are at a high risk of developing dental disease. Pediatricians are the ones with early access to children and thus have an opportunity to directly impact the oral health of the infant [9]. They can evaluate the risks of dental problems and enlighten parents and carers about the prevention of dental disease. Also, they can provide screening services for the early identification of dental problems, give recommendations about the need to see a dental clinic, and refer those who are in need [10]. Therefore, it is necessary to evaluate the degree to which pediatricians are knowledgeable about oral and dental healthcare practices and prevention.

Hence, our study aims to evaluate the level of knowledge, awareness, and practice among the pediatricians in Assam, India, toward infant oral healthcare needs.

## Materials and methods

A questionnaire-based (KAP - Knowledge, Awareness, and Practice) survey of infant oral health care was initiated among the pediatricians of the state of Assam. The study was presented to and approved by the Institutional Ethics Committee of Diphu Medical College, Assam, India, with institutional review board number ICE/DMCH/1.

A comprehensive questionnaire was prepared containing 32 questions (including demographic data and close-ended questions) [11,12] and distributed both electronically and via hard copy among Pediatricians across the state of Assam. The questionnaire was prevalidated by a pediatric dentist with a face value of 0.8. The time given to respond was for a week and reminders were sent every day by mail. A sample of 120 pediatricians was selected and verbal consent was taken. Convenient sampling was used to obtain the data from known pediatricians and those who were easily approachable; the response rate was 92%, whereas the remaining were nonresponsive. The reliability and validity of the questionnaire were checked by doing a pilot study where the kappa value was found to be > 0.80.

Almost more than 200 pediatricians were approached personally, more than the sample size of the study, but many failed to respond. The sample size of 120 was determined for the survey assessing infant oral health care knowledge among pediatricians in Assam using a formula that takes into account the desired confidence level, margin of error, estimated population size, and expected variability in the data. A 95% confidence level and a 5% margin of error were chosen as standard values to ensure a high level of confidence in the survey results while keeping the margin of error acceptable. Since the exact population size of pediatricians in Assam was not available, a conservative estimate was used. The formula, based on these parameters, yielded a sample size of 120 pediatricians. This sample size was considered sufficient to provide statistically meaningful results for the study's objectives while accounting for potential non-responses and variations in responses among the population of pediatricians in Assam.

The selection criteria for this survey included pediatricians practicing within the state of Assam, registered with the Assam State Medical Council, and willing to provide verbal consent for participation. The inclusion criteria encompassed pediatricians who met the geographical, institutional, and consent-related prerequisites. Pediatricians who did not meet the aforementioned inclusion criteria, such as those practicing outside of Assam or those unwilling to provide consent, were excluded from the study. 

The questionnaire was divided into demographic data, knowledge and awareness, and practice to get a comprehensive outlook of the understanding among the pediatricians of Assam regarding infant oral health care in their day-to-day practice. The study was carried out over a period of one year and bi-monthly personal reminders via messages and calls were given to the participants.

Collected data was entered in an Excel spreadsheet (Microsoft Corporation, Redmond, Washington, United States), and IBM SPSS Statistics for Windows, Version 20, (Released 2011; IBM Corp., Armonk, New York, United States) was utilized for analysis. The chi-square test was used to determine the associations between the main outcome variable and other categorical variables.

## Results

Demographics

Out of the 110 participants who responded, 73 were male and 37 were female. A total of 64 (58.2%) were based in urban centers; 29 (26.4%) in semi-urban areas, and 17 (15.4%) in rural areas; 101 were practicing and 9 were non-practicing (Table 1).

**Table 1:** Distribution of study subjects based on sociodemographic variables and knowledge scores. Knowledge scores (0-10): Adequate/Good = 7-10, Fair/Marginal = 4-6, Inadequate/Poor = 0-4

Variables	Number (n)	Percentage
Gender		
Males	73	66.4
Females	37	33.6
Geographic location		
Urban	64	58.2
Semiurban	29	26.4
Rural	17	15.4
Currently practicing		
Yes	101	91.8
No	9	8.2
Number of years of practicing as a pediatrician		
Up to 10 years	90	81.8
10-20 years	15	13.6
More than 20 years	5	4.5
Government/private practice		
Government	94	85.5
Private	16	14.5
Knowledge scores		
Adequate	46	41.8
Fair/marginal	29	26.4
Inadequate	35	31.8

Knowledge/awareness/attitude

The majority of participants 64 (around 58%) claimed to have known that the cavity-causing bacteria can be transmitted between a mother and her child; 85 (77%) of the participants were aware that the initial phase of tooth decay is manifested as a white spot on the tooth surface. Furthermore, almost all the participants were aware of the decrease in salivary flow during night time which makes the tooth more susceptible to decay, making brushing before sleep an essential practice; 87 (79%) were aware of the detrimental effect of bottle feeding at night toward oral health, and all the participants were also aware of the harmful effect of the use of sweetened pacifiers at night, which lead to increased incidence of dental decay. The participants (n=65, 59%) also reflected their awareness that bottle-fed babies are not the only ones to be affected by dental decay. However, around 68 (62%) were unaware that prolonged at-will breastfeeding leads to dental decay. Most of the participants demonstrated awareness of the role of fluoride in the prevention of caries (101, 92%); counseling on feeding and weaning led to a decrease in the incidence of early childhood caries (104, 94%); and that if left untreated, dental decay can affect the general health of the child (110, 100%) (Table 2).

**Table 2:** Distribution of attitude-based responses of the study subjects.

Sl. No.	Questions	Strongly agree n (%)	Agree n (%)	Neither agree nor disagree n (%)	Disagree n (%)	Strongly disagree n (%)
1	Cavity-causing bacteria can be transmitted between mother and child	10 (9.1%)	54 (49.1%)	5 (4.5%)	38 (34.5%)	3 (2.7%)
2	The first signs of tooth decay are the presence of white spots on the tooth surface	24 (21.8%)	61 (55.5%)	17 (15.5%)	8 (7.3%)	0 (0%)
3	Saliva secretion is decreased at night, at which time the teeth are more susceptible to decay; therefore, brushing at night is essential.	56 (50.9%)	54 (49.1%)	0 (0%)	0 (0%)	0 (0%)
4	Bottle feeding at night leads to dental decay	34 (30.9%)	53 (48.2%)	13 (11.8%)	10 (9.1%)	0 (0%)
5	Frequent use of pacifiers sweetened with sugar, honey, and juice is harmful to teeth, especially at night.	59 (53.6%)	51 (46.4%)	0 (0%)	0 (0%)	0 (0%)
6	Only bottle-fed babies are affected by early childhood tooth decay	8 (7.3%)	20 (18.2%)	17 (15.5%)	60 (54.5%)	5 (4.5%)
7	Prolonged and at-will breastfeeding leads to dental decay	5 (4.5%)	20 (18.2%)	17 (15.5%)	44 (40%)	24 (21.8%)
8	Fluoride has a role in the prevention of caries	49 (44.5%)	52 (47.3%)	0 (0%)	7 (6.4%)	2 (1.8%)
9	Counseling on feeding and weaning practice decreases early childhood caries	58 (52.7%)	46 (41.8%)	4 (3.6%)	2 (1.8%)	0 (0%)
10	Untreated dental decay could affect the general health of a child	64 (58.2%)	46 (41.8%)	0 (0%)	0 (0%)	0 (0%)

Practice

Most of the participants were found to give less importance to talking to the guardians of the children regarding the importance of the first dental visits of the children (Table 3).

**Table 3:** Distribution of practice-based responses of the study subjects.

Variables	Number (n)	Percentage
How often do you talk to parents about the infant’s first dental visit?		
All of the time	11	10
Most of the time	30	27.3
Some of the time	55	50
Never	14	12.7
At what age do you recommend a child go for their first dental visit?		
1 year old or less	44	40
2 years old	46	41.8
3 years old	6	5.5
Over 3 years old	14	12.7
At what age do you actually see children for their first visit?		
0-12 months	90	81.8
12-24 months	10	9.1
24-36 months	5	4.5
Over 36 months	5	4.5

A comparison of knowledge-based scores with gender and years of practice of the study subjects (Table 4) states that among 37 female study subjects, 20 (54.1%) had inadequate oral health knowledge scores, whereas only 15 (40.5%) of female study subjects had adequate and two (5.4%) had fair oral health literacy scores. Among 73 male study subjects, a majority of 31 (42.5%) had adequate and 27 (37%) had fair oral health knowledge, and only 15 (20.5%) had inadequate oral health literacy. On comparison of knowledge-based scores of study subjects with years of practice, it was observed that the study subjects with more than 20 years of practice had more adequate (three, 60%) and fair (two, 40%) oral health knowledge when compared to study subjects with less experience of practicing. A highly statistically significant difference was observed between knowledge scores and gender as well as years of practicing with the study subjects (p-value ≥ 0.05). No statistically significant value was observed in the comparison of knowledge-based scores of the study subjects with the type and place of practice (Table 4).

**Table 4:** Comparison of knowledge-based scores with gender and years of practicing of the study subjects. p<0.05 was considered significant

Variable	Adequate n(%)	Marginal/fair n(%)	Inadequate n(%)	p-value
Gender				0.001
Female	15(40.5%)	2(5.4%)	20(54.1%)	
Male	31(42.5%)	27(37%)	15(20.5%)	
Number of years of practicing				
Up to 10 years	41(45.6%)	17(18.9%)	32(35.6%)	0.001
10-20 years	2(13.3%)	10(66.7%)	3(20%)	
More than 20 years	3(60%)	2(40%)	0(0%)	
Type of practice				0.377
Government	37(39.4%)	25(26.6%)	32(34%)	
Private	9(56.2%)	4(25%)	3(18.8%)	
Place of practice				
Rural	6(35.3%)	6(35.3%)	5(29.4%)	0.127
Semiurban	18(62.1%)	5(17.2%)	6(20.7%)	
Urban	22(34.4%)	18(28.1%)	24(37.5%)	

## Discussion

ECC is a multifactorial infectious disease, primarily attributed to four main etiological factors: susceptible host, cariogenic bacteria, fermentable carbohydrate substrate, and time for interaction of these [13]. ECC, though a rampant disease, is easily preventable by taking various measures like early initiation of brushing and oral health care measures, educating mothers about the vertical transmission of *Streptococcus mutans* from mother to child, preventing prolonged at-will breastfeeding and bottle feeding during sleep, etc. [14]. AAPD and American Academy of Pediatrics (AAP) guidelines recommend the first dental visit by the age of 12 months with the establishment of a dental home for children with a high risk of dental caries [15,16]. Parents thus need to be educated from the birth of a child about the importance of oral health practices and steps to be taken to provide a better quality of life for the child.

However, to achieve this goal, pediatricians and other allied healthcare workers who work in close contact with the child need to play a pivotal role. Many surveys have been conducted at various levels nationally in India [1,11,17-21] and abroad [12,22-24] to validate this hypothesis. Most studies have demonstrated that most pediatricians have various levels of knowledge about ECC and the importance of counseling regarding diet and oral hygiene measures; they fail to communicate and train the caregiver of the child regarding the same and recommend a dental visit before the age of one year [22,25]. Our study findings are also along similar lines. A total of 67% of the respondents showed adequate to fair knowledge scores (Table 1). Most of them demonstrated adequate knowledge regarding the first signs of dental decay, i.e., white spot lesions and the vertical transmission of cavity-causing bacteria from mother to child.

Early inspection of the oral cavity can be carried out by the pediatrician while examining the oropharynx and nasopharynx [12]. Years of practice of a clinician significantly increase the knowledge regarding oral health and related practices. Increased dental education in the form of continuing medical education (CME) programs and inclusion in the curriculum of the medical community are needed to educate service providers and bring a comfort level to pediatricians in providing oral health care to this very young population as seen in earlier studies [12,26]. By early detection of kids who need to see a dentist, routine dental screenings can significantly improve the overall oral health of young children [26,27].

Pediatricians in our study recommend the caregivers of the children to visit the dentist at an early age but they fail to educate them of the need and importance of dental health, which can fail to impart the need for visiting a dentist at an early age and can lead to neglect. Hence the inclusion of oral health and related practices in the pediatricians' curriculum and CME programs regarding the same can keep the pediatricians well informed and motivate them to provide proper oral health guidelines to the child and their caregivers and help them to educate them and refer them to the dentist as early as possible to maintain quality of oral health for the child.

This study has several limitations that should be acknowledged when interpreting the findings. First, the relatively small sample size of 110 pediatricians from the state of Assam may not fully represent the diversity of pediatricians in the region, and the results may not be generalized to other geographic areas. Second, the data collected in the study rely on self-reported responses from the participants, which could introduce response bias and overestimation of knowledge and practices related to infant oral health care and ECC. Furthermore, the research did not delve into potential interventions or strategies to address the identified gaps in pediatricians' knowledge and practices. One of the limitations is also that no written materials were distributed to the participants, despite engaging in verbal discussions with individuals we were already familiar with. However, it should be noted that we have the ability to easily provide study materials to pediatricians, who are known to us, in order to effectively convey our messages. A context-specific program approach is required to increase awareness among pediatricians. However, additionally, the inclusion of pediatricians in national programs like the National Oral Health Programme (NOHP) can be made and training can be imparted in the same.

## Conclusions

In conclusion, the study revealed that a majority of pediatricians in the state of Assam displayed adequate knowledge regarding dental caries and its identification. However, there were notable gaps in their awareness of the detrimental effects of bottle feeding and related habits on oral health. Furthermore, a significant proportion of pediatricians failed to educate caregivers about the importance of early dental visits for children. These findings highlight the need for targeted educational interventions and awareness campaigns among pediatricians to enhance their understanding of infant oral health care and ECC prevention, ultimately benefiting the oral health of children in Assam.
